# Clinical impact of the angiosome concept: is it applicable in femoropopliteal bypasses, limb salvage, and wound healing in critical pathologic limb-threatening ischemia?

**DOI:** 10.1590/1806-9282.20252069

**Published:** 2026-06-15

**Authors:** Tereza Mazurova, Ilker Sengul, Demet Sengul, Miloslav Mazur, Daniel Toman, Petr Vavra, Jan Roman, Anton Pelikan, Matej Pekar

**Affiliations:** 1 AGEL Ostrava Vitkovice Hospital, Department of Surgery – Ostrava, Czech Republic.; 2 University of Ostrava, Faculty of Medicine, Department of Surgery – Ostrava, Czech Republic.; 3 Giresun University, Faculty of Medicine, Thyroidology Unit – Giresun, Turkey.; 4 Giresun University, Faculty of Medicine, Division of Endocrine Surgery – Giresun, Turkey.; 5 Giresun University, Faculty of Medicine, Department of General Surgery – Giresun, Turkey.; 6 Giresun University, Faculty of Medicine, Department of Pathology – Giresun, Turkey.; 7 University Hospital Ostrava, Department of Surgery – Ostrava, Czech Republic.; 8 Tomas Bata University, Faculty of Humanities, Department of Health Care Sciences – Zlin, Czech Republic.; 9 Masaryk University, Department of Physiology – Brno, Czech Republic.

**Keywords:** Ischemia, Reperfusion, Extremities, Surgery, Pathology

## Abstract

**INTRODUCTION:**

The global burden of peripheral artery disease is increasing worldwide. A subset of 5% of these patients ultimately reach the final stage with trophic defects, a condition clinically defined as critical limb-threatening ischemia. Revascularization remains the paramount treatment modality offering the greatest potential for limb preservation. We investigated whether performing direct revascularization according to the angiosome concept significantly influences (i) the superior healing of ischemic defects, (ii) optimized limb salvage rates, and (iii) reduced patient mortality in a cohort treated with femoropopliteal bypass.

**METHODS:**

This rigorous prospective clinical cohort study processed data from 143 patients after femoropopliteal bypass procedures performed at the AGEL Ostrava Vítkovice Hospital between January 2016 and December 2021—all patients presented with critical limb-threatening ischemia, specifically Fontaine stage IV. We employed advanced statistical methods, including Kaplan-Meier survival analysis and the Cox regression model, to evaluate the effect of direct revascularization versus indirect revascularization on primary outcomes: defect healing, limb salvage, and patient mortality.

**RESULTS:**

Statistical analysis robustly confirmed a statistically significant benefit of direct revascularization on (i) defect healing (p=0.02), (ii) limb salvage (p=0.02), and (iii) patient mortality (p=0.04). Specifically, direct revascularization significantly reduced amputation rates and improved overall survival compared to indirect revascularization.

**CONCLUSION:**

Despite the controversies in the literature and the absence of dedicated, large-scale, English-language published studies focusing specifically on the application of the angiosome concept in femoropopliteal bypasses, we demonstrated a statistically significant benefit of direct revascularization on defect healing, limb salvage, and mortality in this specific patient cohort. This evidence suggests that the angiosome concept extends its predictive power proximally to the femoropopliteal bypass targets. Practical application: In patients with critical limb-threatening ischemia, where the indication for femoropopliteal bypass is rendered borderline due to advanced local findings (high-risk Wound, Ischemia, and foot Infection classification) or severe overall comorbidity status, surgical intervention should be preferentially reserved for scenarios guaranteeing direct revascularization, as patients derive minimal benefit from non-targeted indirect revascularization in high-risk settings.

## INTRODUCTION

The global health challenge of peripheral artery disease (PAD) affects up to one-fifth of the worldwide population. The incidence is dependent on age and the presence of risk factors for atherosclerosis and is still increasing. An estimated 5% of individuals suffering from PAD will develop critical limb-threatening ischemia (CLTI), manifesting with a trophic defect. Without timely and effective treatment, these patients often undergo major amputation, compounding the high mortality rates associated with this group. Prevalently, reperfusion injury retains its noteworthiness after the event of ischemia-reperfusion. The most crucial treatment is revascularization, which occurs in approximately half of patients. Of this number, roughly a quarter of patients die within a year, and a third of patients end up with a high amputation^1,2^.

Taylor and Palmer introduced the angiosome concept in 1987. This sophisticated anatomical model involves the systematic division of three-dimensional tissue regions and angiosomes according to the specific main artery that supplies the area. In the context of CLTI management, the foot is conventionally divided into six distinct angiosomes^3^. Direct revascularization (DR) is defined technically as the establishment of a distal anastomosis to or above an artery that directly supplies the defect site (the specific angiosome affected) and is patent. Conversely, indirect revascularization (IR) means that blood flow is primarily augmented into a collateralized territory, an artery other than that supplying the defect site, because the primary angiosome-supplying artery is occluded or otherwise unsuitable for bypass.

The current study evaluated the effects of direct and indirect surgical revascularization on the healing of ischemic defects and limb salvage in patients with CLTI, integrating the predictive value of the angiosome theory according to the recently published Wound, Ischemia, and foot Infection (WIfI) classification of defects. Our central hypothesis was to demonstrate that the angiosome concept retains significant utility and determines a measurable, practical benefit for patients undergoing proximal bypass surgery. Clinically, we often find ourselves in a situation in which we have a patient who would be suitable for bypass surgery, but we are hesitant about the indication. This occurs when the patient is too sick (polymorbid) or when there is a bad condition of the defects (a high WIfI score), resulting in the limb being classified at a high risk of amputation, thus generating significant equipoise regarding whether the operation would make sense at all.

## METHODS

### Study design and setting

This study employed a prospective clinical cohort design, focusing on the procedures performed and the statistical analysis of the data. We included 143 consecutive patients who underwent surgical revascularization at AGEL Ostrava Vítkovice Hospital between 2016 and 2021. Using established statistical methods, the results of data from patients with CLTI in Fontaine stage IV after surgical revascularization with femoropopliteal bypass (FPB) were evaluated.

### Data collection and outcomes

We categorized and studied the type of revascularization (DR vs. IR) and evaluated its effect on defect healing, limb salvage, and patient mortality. The individual patient groups were statistically tested. The significance level (p-value, p) was prospectively set at less than 0.05 (5%). Descriptive statistical methods (means, ranges, medians, and standard deviations) were used to process the results. Specifically, analysis of long-term results was performed using the Kaplan-Meier cumulative survival methodology, and survival curves were compared using the Mantel-Cox test (also known as the log-rank test). We used a Cox regression model to determine the effects of the independent risk factors. This study was a prospective, clinical, unicentric, unblinded, and non-randomized trial. Ethical standards, including anonymization of patient data and compliance with GDPR, were rigorously ensured.

### Patient and procedure characteristics

**Figure 1:**
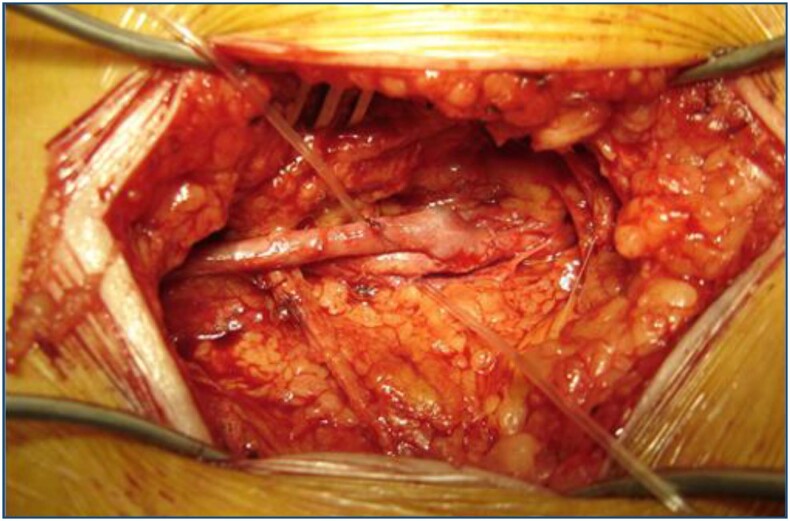
Distal anastomosis of femoropopliteal bypass.

All 143 patients underwent proximal or distal FPB (Figure 1) for CLTI at Fontaine IV. Conduit selection comprised autologous great saphenous veins, polyester, or polytetrafluoroethylene grafts. Each procedure was performed by a vascular surgeon with specialized training in a vascular surgical operating room equipped with an integrated X-ray system that allowed for intraoperative angiography. Of the 143 patients, 84 were men (59%), and 116 were smokers or ex-smokers (81%), and the age ranged from 42 to 88 years, with a median age of 67 years. A significant proportion of 77 patients (54%) had type 2 diabetes mellitus. The follow-up period ranged from 6 to 70 months, with a mean of 29 months. Eight patients were lost to follow-up.

### Inclusion and exclusion criteria

Strict inclusion criteria were applied to compare homogeneous groups of patients in whom other known systemic or local factors affecting the healing process could not influence the outcome of the defect. Therefore, malnourished patients, patients with known immune disorders, patients with poor compliance with treatment, and those with a mixed etiology of defects were systematically excluded. Cases in which the defects were borderline or involved a range of several angiosomes were also excluded to ensure precise classification of DR vs. IR. Additionally, owing to the potential for distortion of the results, patients who underwent endovascular treatment after bypass, which may theoretically convert IR to DR, were excluded.

## RESULTS

The effect of direct and indirect revascularization on healing and healing time of defects

DR was statistically associated with superior healing rates and significantly reduced defect healing times (p=0.02). In the DR group, 50.9% of the defects healed at 3 months post-procedure, compared to 45.1% of the defects in the IR group. Furthermore, at subsequent follow-up intervals of 6, 9, and 12 months, 74.28, 84.57, and 89.6% of defects healed after DR, respectively, compared with 51.2, 62.4, and 71.8% in IR. This demonstrated a marked and sustained advantage in wound closure kinetics for DR.

### The effect of direct and indirect revascularization on limb salvage

**Figure 2:**
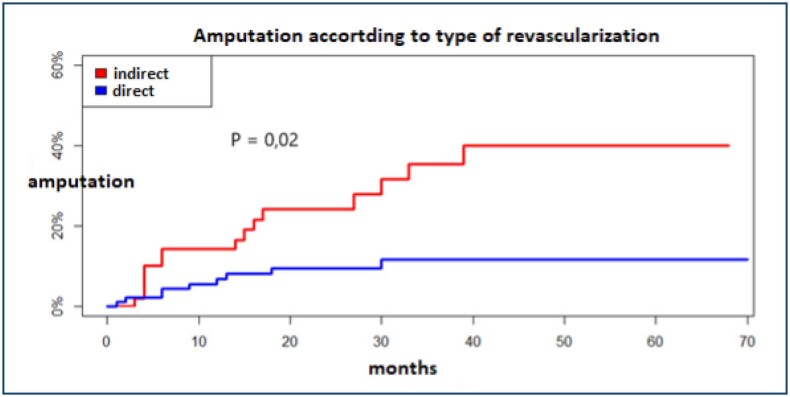
Influence of type of revascularization on "amputation-free survival."

The number of patients requiring major amputation was significantly higher after IR compared to DR (p=0.02). In patients undergoing DR, 2.1% underwent amputation after 3 months, compared with 4.2% in patients with IR at the same time point. Furthermore, examining cumulative amputation rates 6, 9, and 12 months after DR revealed 4.4, 5.5, and 6.8% amputations, respectively, compared to substantially higher rates of 14.2, 15.3, and 16.6% for IR. These data strongly support DR as a critical factor for achieving amputation-free survival in this cohort (Figure 2).

### The effect of direct and indirect revascularization on mortality

**Figure 3:**
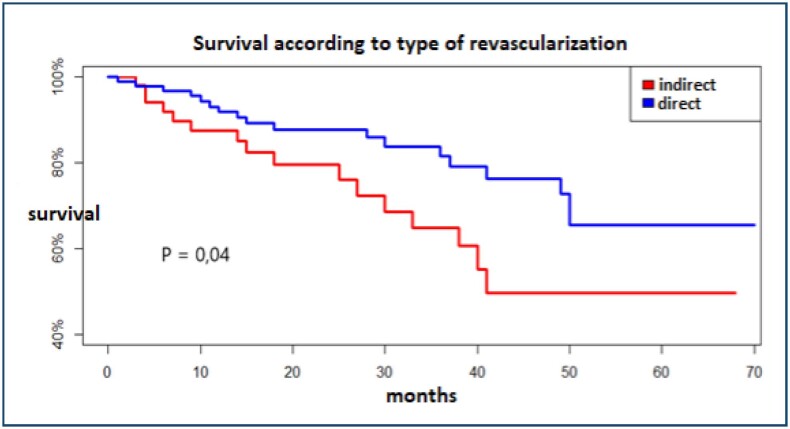
Effect of type of revascularization on mortality.

Patients who were revascularized directly (according to angiosome theory) had a significantly lower mortality rate (p=0.04). In patients undergoing DR, mortality within 3 months was 2.2%, whereas in patients undergoing IR, it was 2%. Furthermore, after 6, 9, and 12 months, the mortality rates in patients with DR were 3.3, 4.5, and 8.2%, respectively, compared with significantly higher rates of 8.1, 12.5, and 13.7%, respectively, in patients with IR. The sustained divergence in the survival curves underscores the long-term systemic benefits associated with successful targeted revascularization (Figure 3).

## DISCUSSION

PAD is a growing concern in the population. The incidence and prevalence have been increasing for several decades, and it does not seem that the increase is expected to slow. Although only a small percentage of these patients suffer from CLTI, it is still a large enough number to consider this diagnosis in detail. These patients typically have a poor prognosis, often end up with high amputation, frequently suffer from depression^4^, and thus, severely burden the health and social system. First, it is essential to focus on primary and secondary prevention, similar to other population-based diseases. However, it is equally important to continually develop and explore new procedures and methods of treatment that are more effective, thereby preserving the quality of life for patients^4-6^.

### Literature review and rationale for the study focus

Previous research on the predictive value of the angiosome concept has yielded ambiguous or contradictory results in some vascular beds. If there was any mention of revascularization according to the angiosome concept in surgical patients in the literature, it was almost exclusively for crural or pedal bypasses (infrapopliteal arteries), which are generally less frequently indicated^7,8^ in routine practice than FPB. Alternatively, existing data were obtained from studies that did not strictly apply angiosome theory for planning or analysis. Given the significant gap in the proximal bypass literature, we decided to focus our work exclusively on patients who underwent the more commonly performed femoropopliteal supragenual or intragenual bypasses. Simultaneously, we included the use of the new WIfI classification to standardize defect severity assessment.

### Clinical implications of findings

We evaluated the key clinical outcomes of healing time of defects, limb salvage, and patient mortality. Prior to analysis, we had no concrete expectations regarding the future outcome, given that no one had specifically processed such data for FPB, and the results of similar distal studies were contradictory^9-11^. Our primary goal was to identify additional objective criteria by which we could more confidently determine that the indication for surgical revascularization is correct in patients with CLTI and that the patient is likely to benefit from it^12^.

The necessity of such criteria arises because the indication for the procedure is often questionable in certain patient subsets. These are patients who are advanced in age, polymorbid, often after cardiac procedures, or have carotid artery stenosis. In such fragile patients, we hesitate about whether revascularization will bring the desired effect, whether they will undergo an unnecessarily large operation with an uncertain outcome for the limb, and, moreover, whether they will face the risks of higher perioperative morbidity and mortality^13^.

Regarding the treatment of PAD, it is crucial to follow new findings regarding pharmacotherapy and revascularization procedures^14,15^. However, another major uncertainty is encountered in patients who already have large or infected trophic defects (reflecting a high WIfI score). In these patients, despite careful necrotectomy and further local care, there is always a higher risk of complications, a higher risk of bypass closure, subsequent infection, and ultimately treatment failure. In these severe cases, a direct high amputation of the limb is often more likely to be considered.

## CONCLUSION

The results of our study provide compelling evidence, confirming the usefulness of revascularization according to the angiosome concept by demonstrating a statistically significant dependence of DR on defect healing, limb salvage, and even overall patient mortality. Quantitatively, almost three times as many patients who underwent DR healed within one year as those who underwent IR.

Following DR, there were more than 50% fewer major amputations within one year than in the group of patients with IR, and as for mortality, half as many patients died within one year after DR than after IR. This strong correlation supports our consideration of the importance of the angiosome concept, especially in cases where the clinical indication for surgical revascularization is marginal, either due to advanced local findings (WIfI score) or the severe general condition of the patient, placing them at the edge of the indication.

In essence, we confirmed the importance of the angiosome concept in performing FPBs, suggesting that surgical planning in patients with CLTI is mandatory.

## Data Availability

The datasets generated and/or analyzed during the current study are available from the corresponding author upon reasonable request.

## References

[1] Goodney P, Shah S, Hu YD, Suckow B, Kinlay S, Armstrong DG (2022). A systematic review of patient-reported outcome measures patients with chronic limb-threatening ischemia. J Vasc Surg.

[2] Sengul I, Sengul D (2021). Clinical insights on pre- and novel postconditioning in equine jejunal ischemia. Med Arch.

[3] Deguchi J (2020). Angiosome~from the standpoint of bypass surgery. Ann Vasc Dis.

[4] Zielke T, Korepta L, Wesolowski M, D‘Andrea M, Aulivola B (2024). The association of comorbid depression with mortality and amputation risk in patients with chronic limb-threatening ischemia. J Vasc Surg.

[5] Cattermole TC, Schimmel ML, Carpenter RL, Callas PW, Gramling R, Bertges DJ (2023). Integration of palliative care consultation into the management of patients with chronic limb-threatening ischemia. J Vasc Surg.

[6] Conte MS, Bradbury AW, Kolh P, White JV, Dick F, Fitridge R (2019). Global vascular guidelines on the management of chronic limb-threatening ischemia. Eur J Vasc Endovasc Surg.

[7] Dilaver N, Twine CP, Bosanquet DC (2018). Editor‘s choice - direct vs. indirect angiosomal revascularisation of infrapopliteal arteries, an updated systematic review and meta-analysis. Eur J Vasc Endovasc Surg.

[8] Jongsma H, Bekken JA, Akkersdijk GP, Hoeks SE, Verhagen HJ, Fioole B (2017). Angiosome-directed revascularization in patients with critical limb ischemia. J Vasc Surg.

[9] Neville RF, Attinger CE, Bulan EJ, Ducic I, Thomassen M, Sidawy AN (2009). Revascularization of a specific angiosome for limb salvage: does the target artery matter?. Ann Vasc Surg.

[10] Ricco JB, Gargiulo M, Stella A, Abualhin M, Gallitto E, Desvergnes M (2017). Impact of angiosome- and nonangiosome-targeted peroneal bypass on limb salvage and healing in patients with chronic limb-threatening ischemia. J Vasc Surg.

[11] Azuma N, Uchida H, Kokubo T, Koya A, Akasaka N, Sasajima T (2012). Factors influencing wound healing of critical ischaemic foot after bypass surgery: is the angiosome important in selecting bypass target artery?. Eur J Vasc Endovasc Surg.

[12] Betz T, Ingolf T, Markus S, Florian Z, Christian U (2022). Evaluation of long-term outcomes of femoropopliteal bypass surgery in patients with chronic limb-threatening ischemia in an endovascular era. Ann Vasc Surg.

[13] Casajuana Urgell E, Calsina Juscafresa L, Nieto Fernandez L, Romero Montaña L, Llort Pont C, Clarà Velasco A (2022). Critical limb ischemia in nonagenarians: a challenge of our times. World J Surg.

[14] Mazurová T, Sengul I, Toman D, Pelikán A, Sengul D, Mazur M (2022). Endofibrosis as a causative agent of the peripheral artery disease: a report of two cases for professional cyclists. Cureus.

[15] Mazurová T, Sengul I, Toman D, Pelikán A, Sengul D, Mazur M (2023). Endofibrosis as a cause of peripheral artery disease: a comprehensive review and proposal of two novel algorithms for diagnosis and treatment. Rev Assoc Med Bras (1992).

